# An Integrated Analysis of the Identified PRPF19 as an Onco-immunological Biomarker Encompassing the Tumor Microenvironment, Disease Progression, and Prognoses in Hepatocellular Carcinoma

**DOI:** 10.3389/fcell.2022.840010

**Published:** 2022-02-17

**Authors:** Ming Yang, Yiwen Qiu, Yi Yang, Wentao Wang

**Affiliations:** Department of Liver Surgery, West China Hospital, Sichuan University, Chengdu, China

**Keywords:** Prpf19, tumor-infiltrating immune cell, biomarker, prognoses, liver cancer

## Abstract

**Background:** Targeting the mRNA splicing process has been identified as a therapeutic strategy for human cancer. PRPF19 is an RNA binding protein that is involved in pre-mRNA processing and repairing DNA damage; the aberrant expression of PRPF19 is potentially associated with carcinogenesis. However, the biological role of PRPF19 in hepatocellular carcinoma (HCC) is still elusive.

**Methods:** Data obtained from TCGA, Oncomine, and GEO were used to investigate the PRPF19 expression level and its role in tumor immune infiltration, prognosis, and the tumor progression of cohorts from HCC. Using various databases and tools (UALCAN, TIMER, TISMO, and PathCards), we presented the potential mechanisms of PFPF19 upregulation, PRPF19-related pathways, and its biological functions in liver cancer.

**Results:** For HCC, PRPF19 expression was found upregulated both in single tumor cells and tissues. Furthermore, the increased expression of PRPF19 was significantly correlated to clinical characteristics: advanced stage, vascular invasion, high AFP, and poor prognosis of HCC. According to the tumor-immunological analysis, we found that PRPF19 is positively correlated with infiltrating myeloid-derived suppressor cells (MDSCs). Moreover, the microenvironment of HCC tissues with high expression of PRPF19 is highly immunosuppressive (lower T-lymphocytes, multiple immune checkpoints upregulated). Patients with high expression of PRPF19 and high MDSCs had a worse survival prognosis as well. TP53 mutation may have a positive effect on PRPF19 expression *via* decreased promoter methylation of PRPF19. By TF-mRNA network analysis, key transcription factors (TFs) in TC-NER and PCS pathways (PRPF19 involved) were identified.

**Conclusion:** This work implied that PRPF19 is associated with tumor immune evasion and progression, and serves as a prognostic marker for worse clinical outcomes with HCC. Thus, this critical regulator could serve as a potential therapeutic target of HCC.

## Introduction

Liver hepatocellular carcinoma (LIHC) is considered to be one of the common solid cancers and ranks third in the list of causes of cancer-related deaths worldwide on the basis of the International Agency for Research on Cancer (IARC) statistics reported in 2021 (Siegel et al., 2021). LIHC is characterized by high malignancy, poor prognosis, and high recurrence rates worldwide. The main reasons for the high mortality of LIHC are difficulty in early detection, lack of effective treatments, and extremely high rates of intrahepatic metastasis (Villanueva, 2019). Molecularly targeted therapy is an active area of drug development in cancer therapy. However, existing targeted drugs typically induce an incomplete tumor response that is followed by drug resistance in advanced-stage LIHC patients (Bruix et al., 2019). Given the high mortality and unsatisfactory treatment options in LIHC, identifying new LIHC-characteristic markers for patient stratification and therapeutic intervention is a key unmet medical need in this disease.

Aside from acting as a messenger and translator of the genetic information in the cell, mRNA itself also exerts roles in regulating protein expression and modulating cellular physiology (Ju et al., 2019). In eukaryotic cells, transforming the mRNA precursor molecule (pre-mRNA) to mature mRNA is an essential step in gene expression. Abnormal pre-mRNA splicing regulation has been causally related to the development of tumors (Baralle and Giudice, 2017). In acute myeloid leukemia (AML) patients with IDH mutations, frequent mutations in splicing factor mutations are shown including SRSF2 and SF3B1 (Ohgami et al., 2015). In addition, antineoplastic biological agents targeting specific spliceosomal molecular mechanisms are also currently in development (Yoshimi and Abdel-Wahab, 2017). However, few studies have reported on the splicing factors as a prognostic marker or therapeutic target in LIHC.

Pre-mRNA processing factor 19 (PRPF19) is one of the altered splicing factors, which are essential for cell survival and DNA repair (Beck et al., 2008). PRPF19 is located between q12.1 and q13.1 in chromosome 11. Indeed, PRPF19 aberrant expressed in multiple human cancers has been previously reported in literature. Yihong et al. explored a potential mechanism about PRPF19 in tongue cancer growth and chemoradiotherapy resistance (He et al., 2021). Huang et al. found that PRPF19 participated in mitotic progression and arrests cell cycle in hepatocellular carcinoma (HCC) cells (Huang et al., 2017). Yin et al. also discovered that aberrant PRPF19 is associated with the event of vascular invasion in HCC patients (Yin et al., 2016). It was reported recently by Yuanxia et al. (Cai et al., 2020) that high expression of PRPF19 promotes invasion, migration, and EMT in neuroblastoma. However, to our knowledge, the PRPF19 gene in HCC has not been systematically evaluated and warrants further research.

Here, intending to assess the clinical value of PRPF19 in liver cancer, we performed a multi-dimensional analysis of integrated tumor immunity, somatic mutation, and functional networks to capture multiple aspects of information of PRPF19 in LIHC. These findings reveal the potential of PRPF19 as a novel therapeutic target for HCC treatment and prognosis assessment.

## Materials and Methods

### PRPF19 Expression in Liver Cancer

The RNA high-throughput sequencing data of the hepatocellular cancer cases used in this study were from the following three databases: The Cancer Genome Atlas (TCGA) portal (https://tcga-data.nci.nih.gov), NCBI’s Gene Expression Omnibus (GEO) (Edgar et al., 2002), and the Oncomine website (http://www.oncomine.com). PRPF19 expression data in all samples were extracted for subsequent analysis and visualization using R programming language version 4.1.0. The visualization of PRPF19 differential expression in normal and tumor tissues was completed and generated using the R library gglot2.

### PRPF19 Clinical Correlations in Liver Cancer

We downloaded the clinicopathological data of hepatocellular cancer cases from the TCGA. Samples with full clinicopathological data including age, sex, grade, TNM stage, microvascular invasion, fibrosis Ishak score, and the alpha-fetoprotein level were grouped based on the high and low expression of PRPF19. Univariate logistic regression was performed to analyze the correlations between PRPF19 expression and clinical variables with the binary outcome.

### PRPF19 Prognosis Value in Liver Cancer

To assess the relationship between the PRPF19 expression and survival of liver cancer, the Kaplan–Meier method was selected to calculate the median for time-to-event endpoints, which reported the corresponding 95% CIs. Clinical outcome metrics of overall survival (OS), progression-free interval (PFI), disease-free interval (DFI), and disease-specific survival (DSS) were derived from the TCGA-LIHC cohort. The “surv-cutpoint” function of the survminer R package was used to investigate the optimal cutoff for dividing high and low expression samples (Lauria et al., 2020). Subgroup analyses were also carried out to evaluate the effect of PRPF19 prognosis on OS across various subgroups. Patients were stratified into different subgroups according to the tumor-node-metastasis (TNM) stage and microvascular invasion status. Then, the prognostic value of PRPF19 expression was estimated by Kaplan–Meier survival curves, and the log-rank test was utilized to test the significance among different survival curves.

### Tumor Immunology Analysis of PRPF19

The correlation analysis of the 24 immune cell scores with PRPF19 expression was first performed. Single-sample GSEA (ssGSEA) was utilized for immune deconvolution analyses to quantify the enrichment levels of immune cells (Sanchez et al., 2020). TISMO (tismo.cistrome.org) database was used for comparing the response to immune-checkpoint blockers (ICBs) of syngeneic mouse models with different expression levels of PRPF19. Analysis of the co-expression of immune-related genes including the “ICs” and “immunosuppressive molecules” and PRPF19 was also performed for the potential role in the tumor microenvironment of PRPF19. Furthermore, the relationship between PRPF19 expression and infiltrate level of MDSCs in LIHC tissues was evaluated including correlation and survival analysis, based on the Tumor Immune Estimation Resource (TIMER) online database (cistrome.shinyapps.io/timer). Also, the marker genes of MDSCs and PRPF19 expression correlation analysis were conducted.

### Potential Regulatory Mechanisms of PRPF19

PathCards is an integrated database of human biological pathways (Belinky et al., 2015). For analysis of the intermolecular interaction, we extracted PRPF19-related gene sets from the PathCards database. In this study, the top 100 genes in correlation with PRPF19 in LIHC were also extracted to take the intersection with the aforementioned PRPF19-related genes in humans. The miRNA–PRPF19–related protein and gene network was generated from the JASPAR database *via* the NetworkAnalyst website (Zhou et al., 2019).

### Liver Tissue Specimen Collection and qRT-PCR

LIHC tissues and adjacent normal liver tissues were collected from surgical samples in West China Hospital, Sichuan University, Chengdu, China. The protocol and sample size (30 pairs) of this study was approved by the Ethics Committee of West China Hospital, Sichuan University. Total RNA of all clinical samples was extracted using the TRIzol reagent (BoMei Biotechnology Co. Ltd., HeFei, China). Then, we conducted synthesizing of first-strand cDNA and real-time PCR, respectively. The following primer sequences for this assay were used: PRPF19 (forward): 5′-ATC​TGC​TCC​ATC​TCT​AAC​GAA​G-3′ and (reverse): 5′-TAC​CAT​TCT​CCG​CAA​TGT​ACT​T-3′; ACTB (forward): 5′-CGA​TCC​GCC​GCC​CGT​CCA​CA-3′ and (reverse): 5′-ACG​CAG​CTC​ATT​GTA​GAA​GGG​TGG​TG-3′.

### Statistical Analysis

All statistical analyses were performed using R software (version 4.1.0). Student’s t-test (two-tailed) was conducted in contrast between two groups. Gene expression correlations were analyzed by the Spearman correlation. Results with *p* < 0.05 were considered statistically significant.

## Results

### PRPF19 is Upregulated in Liver Cancer

**FIGURE 1:**
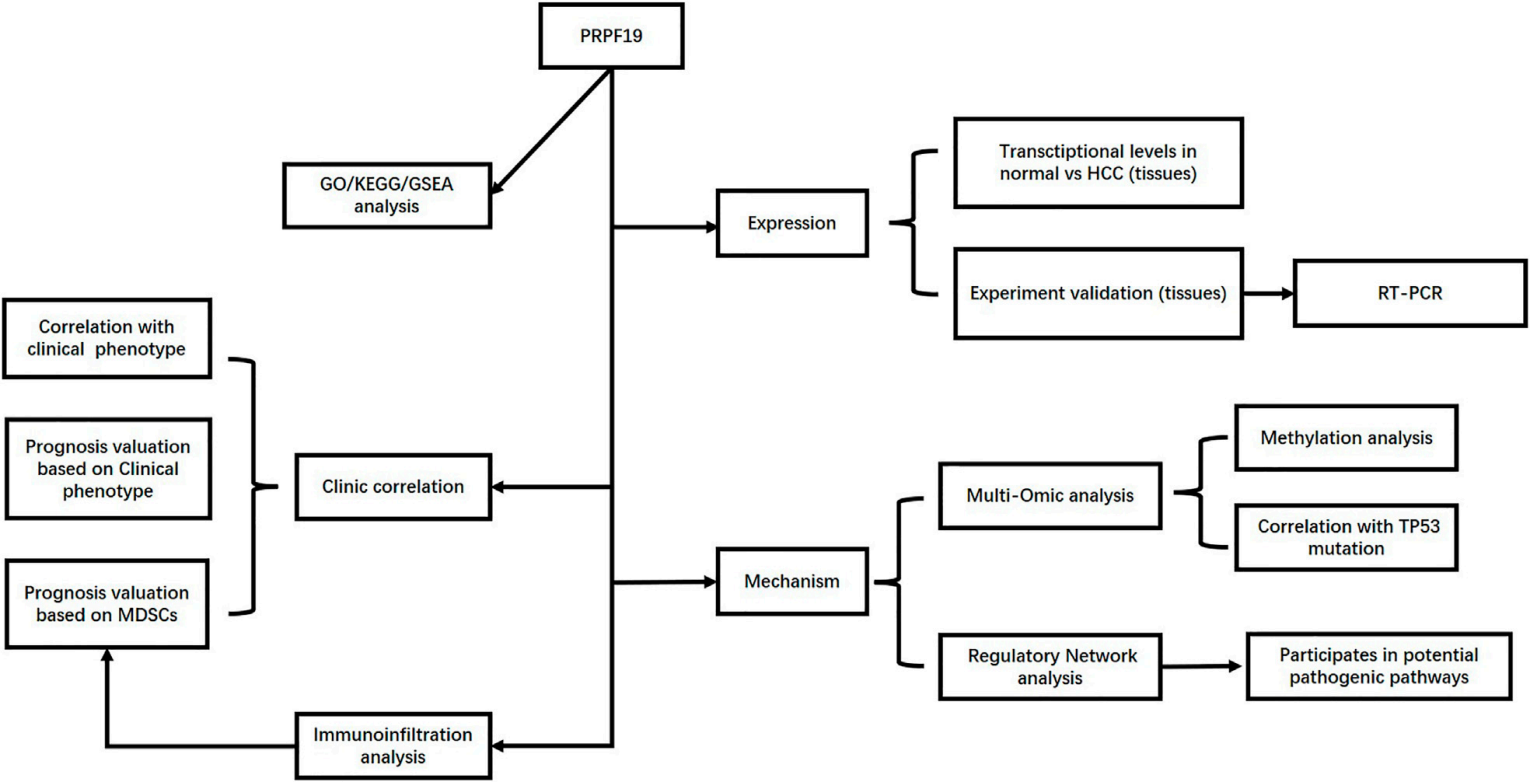
Flow diagram of data collection and method implementation in this work.

**FIGURE 2:**
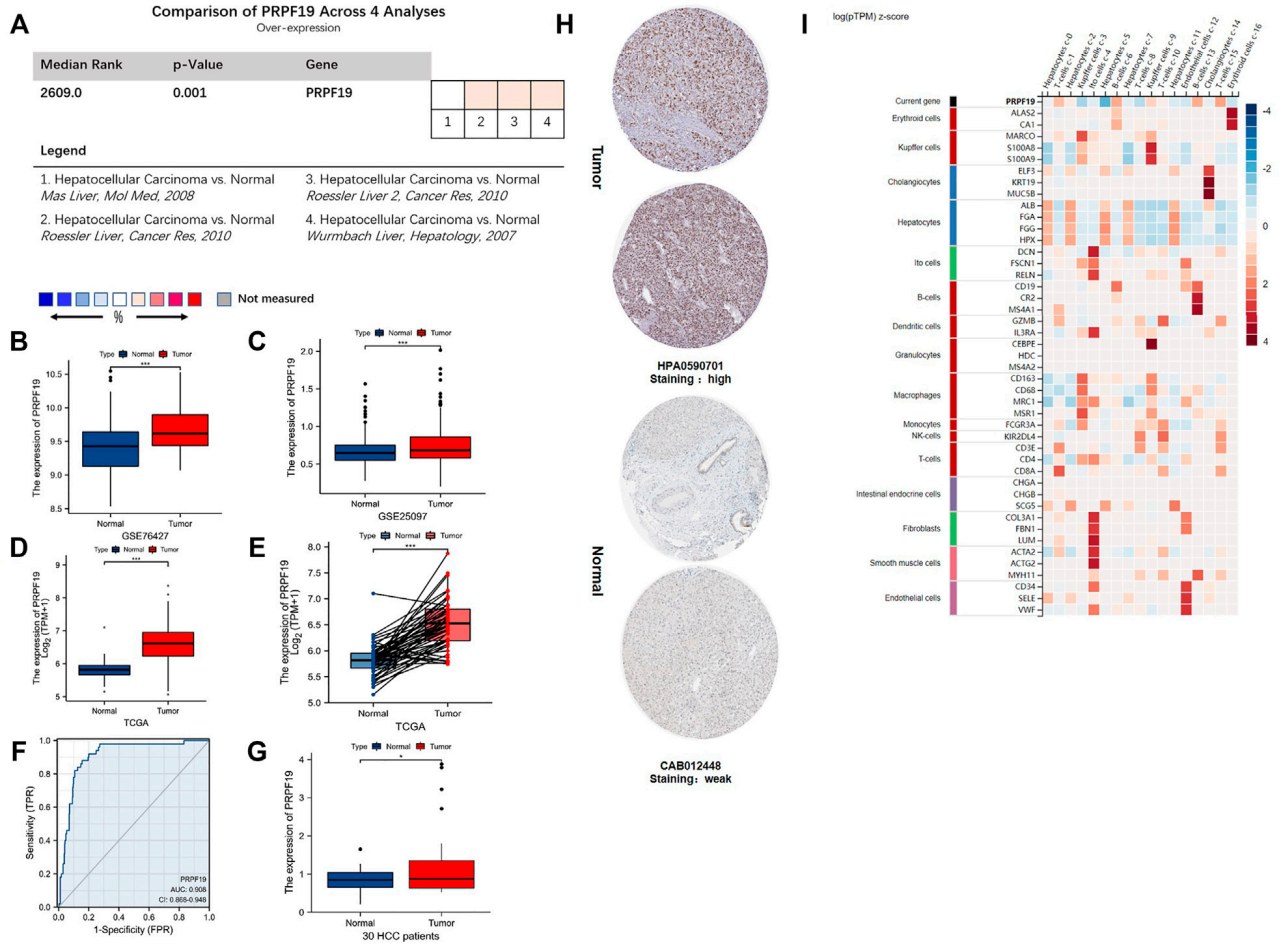
The mRNA and protein expression of PRPF19 in liver cancer. **(A–C)** Overexpression of PRPF19 was found at the Oncomine database and the GEO database series including GSE76427 and GSE25097. **(D)** PRPF19 expression in tumor and normal tissues in liver cancer (LIHC) from the TCGA database. **(E)** PRPF19 expression in indicated paired tumor and normal tissues in LIHC data of TCGA. Black lines connect paired tissues. **(F)** ROC analysis of PRPF19 shows promising discrimination power between tumor and normal tissues. **(G)** PRPF19 expression levels were measured in humans through qRT-PCR. **(H)** Representative immunohistochemistry images of PRPF19 protein expression levels in LIHC and non-cancerous liver tissues derived from the HPA database. **(I)** PRPF19 expression heat map in each cell existed in normal liver tissues (HPA). Data shown as mean ± SD. *p < 0.05, **p < 0.01, ***p < 0.001, ****p < 0.0001.

**FIGURE 3:**
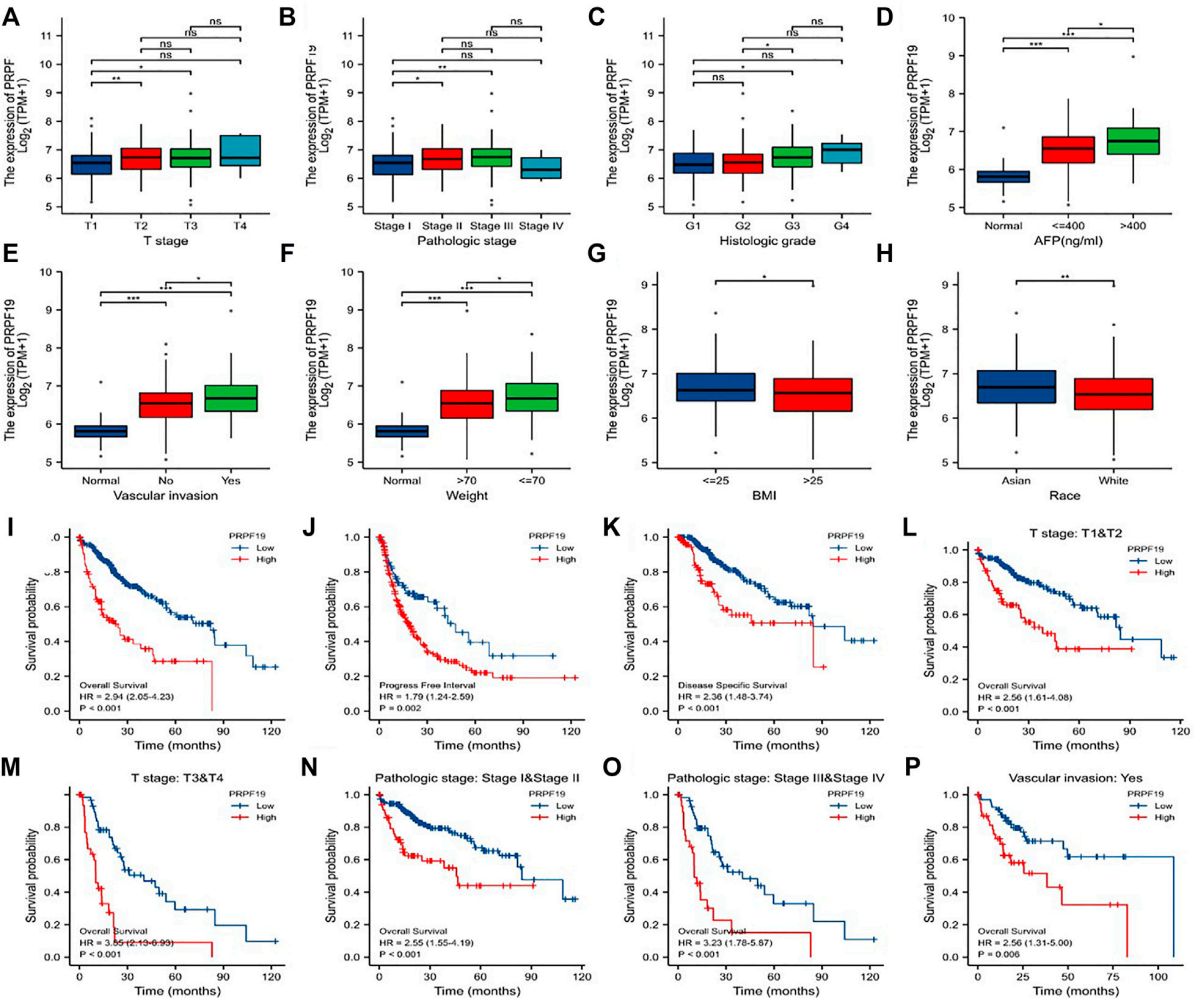
Association of PRPF19 expression with clinicopathologic characteristics of LIHC (TCGA). **(A)** T stage. **(B)** Pathologic stage. **(C)** Histologic grade. **(D)** AFP level. **(E)** Vascular invasion. **(F)** Patients’ weight. **(G)** BMI. **(H)** Race. **(I–K)** LIHC patients with higher expressions of PRPF19 have shorter OS, PFI, DSS compared to those with lower expressions of PRPF19. **(L–P)** Patients with higher PRPF19 have poor OS in T stage, pathologic stage, and vascular invasion subgroups. *p < 0.05, **p < 0.01, ***p < 0.001.

The flow chart for this study is shown in Figure 1. To annotate the expression of PRPF19 in normal liver tissues and liver cancer, we integrated single-cell sequencing (scRNA-seq) data from the Human Protein Atlas (HPA). First, PRPF19 expressed at a lower level in normal hepatocytes c-0/2/5/7/11 was compared with other marker genes of liver cells (Figure 2I). Given the aforementioned observations, we then conducted a more detailed analysis of PRPF19 involved in liver cancer using RNA sequencing data. Figures 2A–E show the transcription levels of the PRPF19 in hepatocellular carcinoma. Meta-analysis of four datasets from Oncomine showed that the median rank was 2,609.0 (*p* = 0.001). Overexpression of PRPF19 was reported by Roessler Liver 1 and 2, Wurmbach Liver except for the Mas liver. Next, we observed that the expression level of PRPF19 was significantly upregulated in patients with LIHC based on the sequencing data of TCGA-LIHC, GSE76427, and GSE25097. A higher protein level of PRPF19 was also detected in the tissue of liver cancer compared to normal liver, as the immunohistochemical sections were represented from HPA datasets. (Figure 3H). Furthermore, setting the expression value of PRPF19 as the variable, the ROC classifier shown in Figure 3F had a high discrimination ability to identify LIHC (AUC = 0.908). Finally, we validated the expression of PRPF19 using 30 paired samples from liver cancer patients. The results revealed that PRPF19 was highly expressed in liver cancer tissues (Figure 2G).

### Clinical Phenotype of Liver Cancer With High Expression PRPF19

The clinical features of PRPF19 are summarized in Figures 3A–H, including the PRPF19 expression analyses according to different T stages, pathologic stages, histologic grade, AFP level, vascular invasion, and weight. Increasing expressed PRPF19 was observed in patients with high AFP levels (>400 ng/ml) and vascular invasion. In addition, the expression of PRPF19 had a negative association with body mass index (BMI) and weight (those with BMI ≤25 and weight ≤70 expressed more than those with BMI>25 and weight>70). When stratified by race, the PRPF19 expression level was elevated across the Asian cases than white populations. We also performed further logistic analyses to determine the correlation between PRPF19 expression and clinicopathological characteristics (Table 1). The results demonstrated that the PRPF19 expression had prominently positive correlations with T stages, pathologic stage, histologic grade, AFP, fibrosis Ishak score, and vascular invasion (*p* < 0.005).

**TABLE 1:** Logistic regression analysis of association between clinicopathological characteristics and PRPF19 expression in LIHC patients.

Characteristics	Odds ratio(OR)	*p* Value
T stage (T1&T2 *vs*. T3&T4)	1.723 (1.073–2.793)	0.025
N stage (N0 *vs*. N1)	2.687 (0.339–54.709)	0.395
M stage (M0 *vs*. M1)	0.914 (0.108–7.710)	0.929
Pathologic stage (Stage I & Stage II vs. Stage IV & Stage III)	1.891 (1.164–3.104)	0.011
Histologic grade (G1&G2 *vs*. G4&G3)	2.111 (1.375–3.266)	<0.001
AFP (ng/ml) (≤400 *vs*. >400)	2.396 (1.358–4.313)	0.003
Fibrosis Ishak score (1/2&0 *vs*. 5/6&3/4)	1.954 (1.136–3.388)	0.016
Vascular invasion (No *vs*. Yes)	1.931 (1.212–3.100)	0.006

Next, the differences within survival in OS, DSS, and PFI were determined by KM curves for investigating the prognostic performance of the PRPF19 in LIHC. As shown in Figures 3I–P, overexpression of PRPF19 was significantly associated with worse OS (*HR* = 2.94), PFI (*HR* = 1.79), and DSS (*HR* = 2.36). Moreover, patients with high expression of PRPF19 had shorter median OS for the T stage, pathologic stage, and vascular invasion subgroups. Together, these results suggested that high expression of PRPF19 is a poor prognostic factor in liver cancer (Figure 3I–P).

### PRPF19 Presented as an Immune-Related Gene in Liver Cancer

The infiltrating level of 24 immune cell types in LIHC including 12 T-lymphocyte subsets, 4 monocyte-derived DCs, 3 natural killer cells, and 5 other immune cells (neutrophils, mast cell, macrophages, eosinophils, and B cell) were calculated using ssGSEA algorithm. As illustrated in Figure 5A, PRPF19 was negatively correlated with CD8 T cells, cytotoxic cells, DC, mast cells, neutrophils, NK cells, pDC, T cells, Tgd, Th17 cells, Th2 cells, and Treg, while it was positively related to the abundance of NK CD56bright cells, T helper cells, and Th2 cells.

**FIGURE 4:**
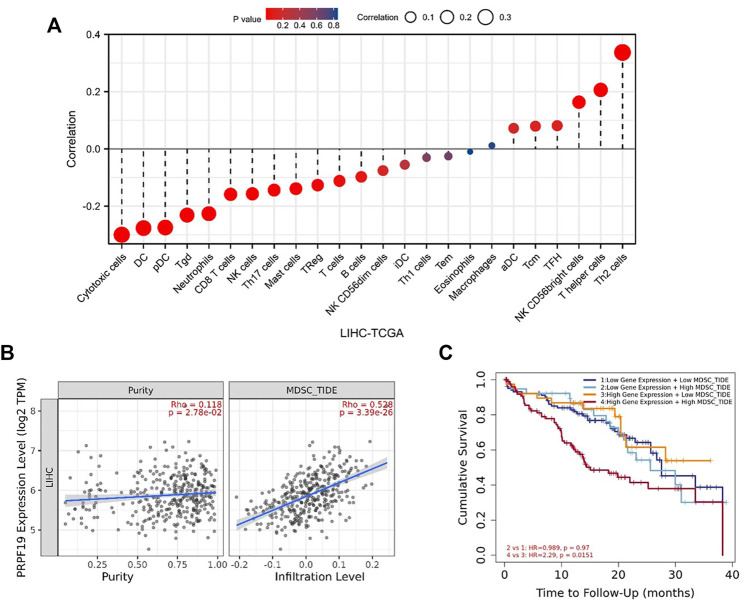
Correlation analysis of PRPF19 level and immune microenvironment in LIHC. **(A)** PRPF19 expression in LIHC tissues negatively correlates with 17 immune cell types. **(B)** Correlation between PRPF19 and infiltrated myeloid-derived suppressor cells (MDSC) in LIHC. **(C)** KM curves according PRPF19 expression and MDSC infiltrating level in LIHC. Data shown as mean ± SD. *p < 0.05, **p < 0.01, ***p < 0.001.

We hypothesized that elevated PRPF19 expression may influence antitumor immunity by recruiting immunosuppressive cell population in the tumor microenvironment. Myeloid-derived suppressor cells (MDSCs) are highly immunosuppressive in the tumor microenvironment and make tumor cells resistant to immunotherapy (Sharma et al., 2017). Next, we further explored the relationship between MDSCs and PRPF19 in liver cancer. The tumor immune dysfunction and rejection (TIDE) algorithm can comprehensively evaluate the infiltration of immunosuppressive suppressor cells in the tumor microenvironment (Jiang et al., 2018). The TIDE algorithm based on the TIMER database was used to assess the MDSC abundance in LIHC. As expected, tumor purity and the infiltration level of the MDSCs were accompanied by elevated PRPF19. Interestingly, when the prognostic analysis was focused on overexpression of PRPF19, those with high MDSC tumors had the worst prognosis compared to those with low MDSC tumors (HR = 2.29 *p* < 0.05) (Figures 4B,C).

**FIGURE 5:**
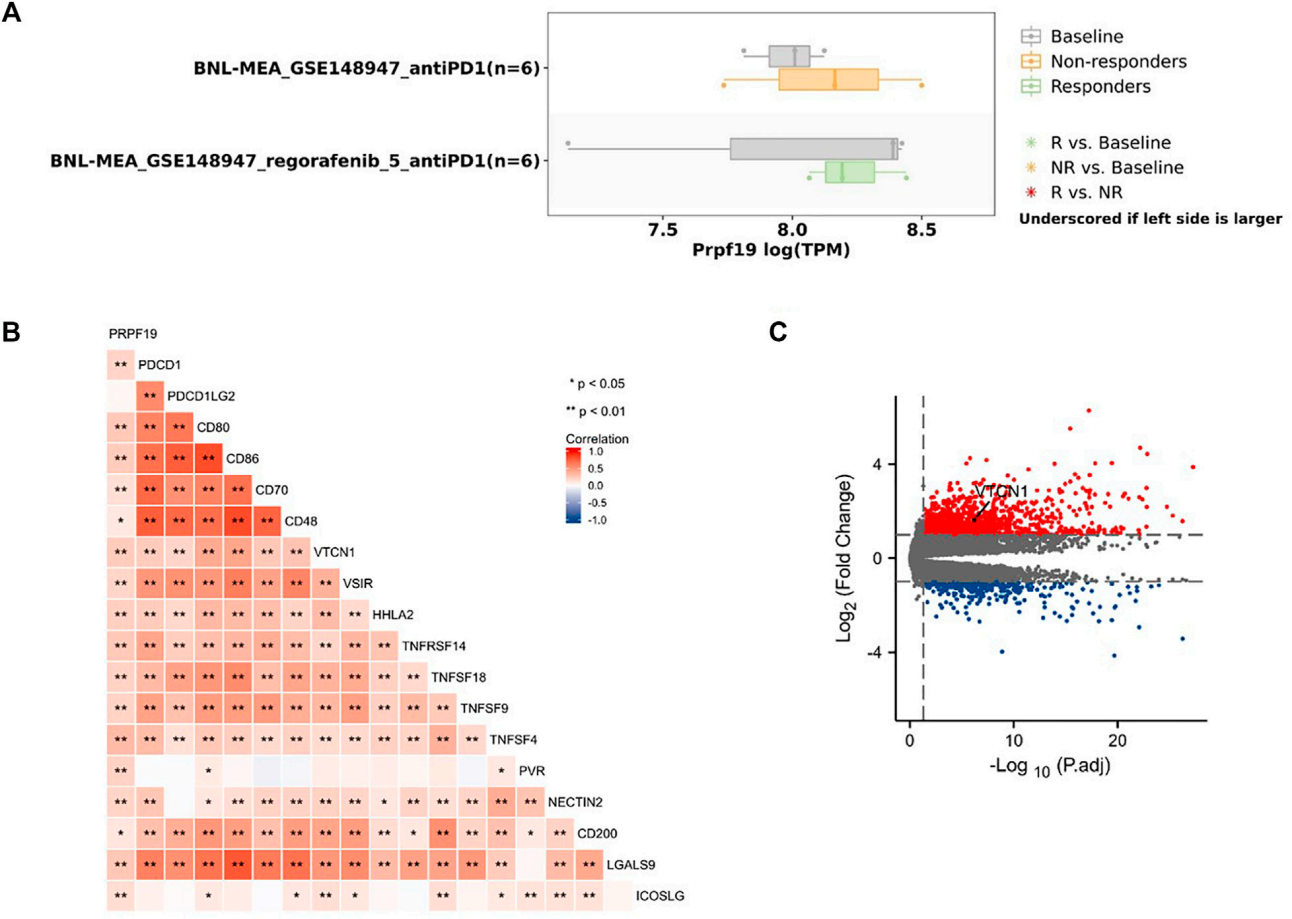
PRPF19 might has a significant effect on the efficacy of LIHC immunotherapy. **(A)** The same trend of PRPF19 expression pattern was seen with poor ICB responsive cell lines base on syngeneic mouse model (TISMO). **(B)** Heat map of the correlation between PRPF19 expression and immune checkpoint genes. **(C)** Volcano plot showing the significantly immunosuppressive gene, VTCN1, in patients with high PRPF19 expression compared with patients with low PRPF19 expression.

To investigate whether the expression pattern of PRPF19 affects immunotherapy, we first inquired TISMO (tismo.cistrome.org) database which collected 1,518 syngeneic mouse model data receiving ICB treatment. Analysis of two cell line data (BNL-MEA) showed that the upregulated PRPF19 related to a poor response for ICB, and the downregulated PRPF19 related to a better response for ICB when compared with maintaining the baseline expression level of PRPF19. The heat map depicts the correlation between the 18 classical immune checkpoints and PRPF19. Among them, PRPF19 was positively correlated with 17 ICIs except PDCD1LG2. Moreover, we observed that overexpression of PRPF19 in LIHC is accompanied by a marked elevation of VTCN1. This trend did not appear in the remaining ICIs. The research showing that blockade of VTCN1 signaling pathway can inhibit the MDSC induces dysfunction of antitumor CD8^+^ T cells (Li et al., 2018). These results identified that PRPF19 may be a possible new target for HCC immunotherapy (Figure 5).

### Construction of miRNA-PRPF19 and Co-expression of Gene Network in Liver Cancer

**FIGURE 6:**
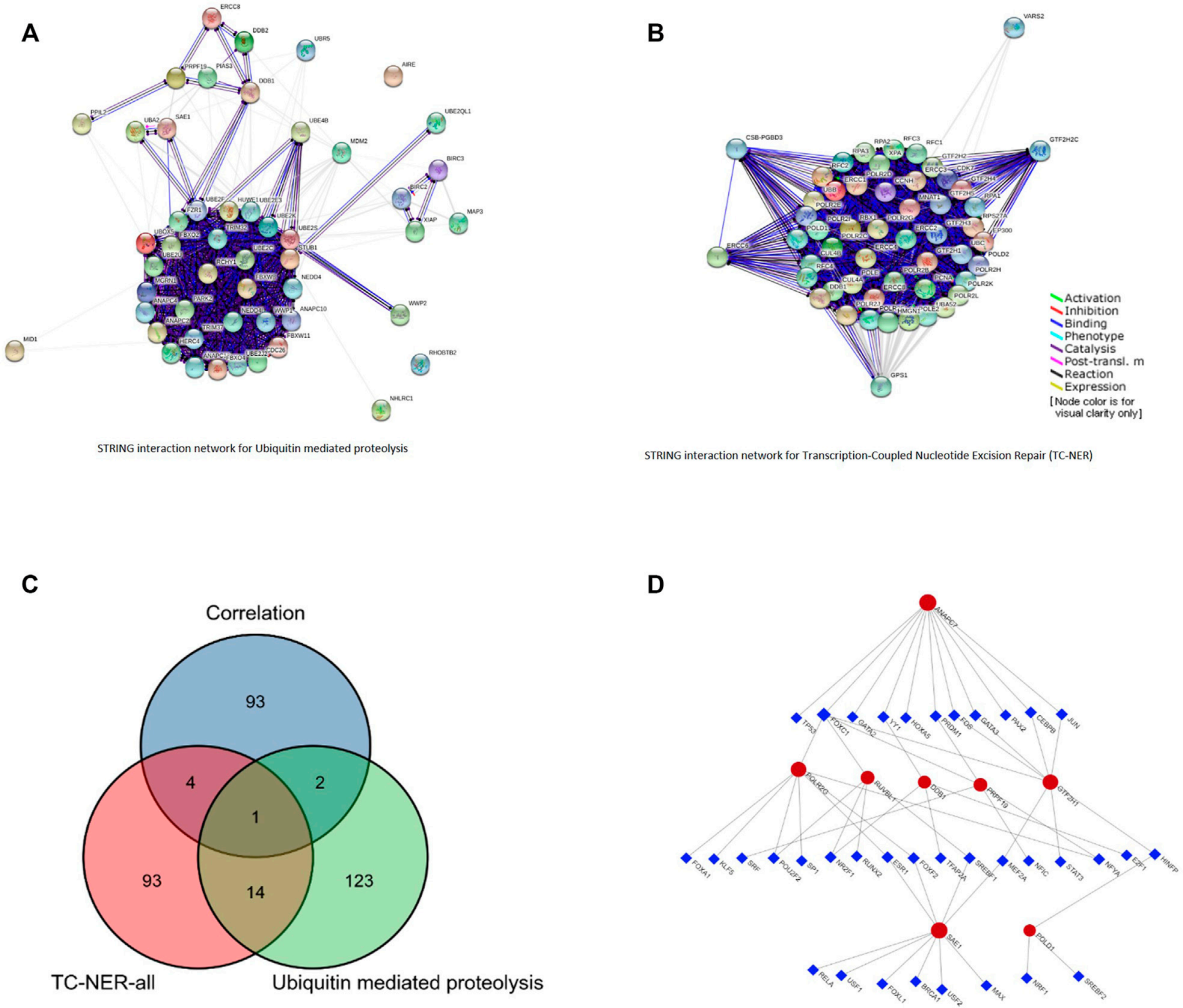
Pathway network for PRPF19-related. **(A)** The interaction network of ubiquitin-mediated proteolysis. **(B)** The interaction network of transcription-coupled nucleotide excision repair (TC-NER). **(C)** The interaction network of 100 positive correlated genes, 112 genes in ubiquitin-mediated proteolysis pathway, and 140 genes in TC-NER pathway. **(D)** TF–eight network generated by NetworkAnalyst software. The regulation network of 36 TFs and 8 mRNAs.

PRPF19 involved two key carcinogenic pathways, including ubiquitin-mediated proteolysis (Martinez-Jiménez et al., 2020) and the transcription-coupled nucleotide excision repair (TC-NER) (Xu et al., 2007). We collected 112 genes and 140 genes respectively at work in the aforementioned pathways from PathCards (pathcards.genecards.org). By taking the intersection of the top 100 PRPF19 expression–related genes and the 252 genes, POLR2G, RUVBL1, POLD1, GTF2H1, SAE1, ANAPC7, DDB1 were selected (Figures 6A–C). Next, we predicted that 36 transcription factors (TFs) targeted the aforementioned seven genes and PRPF19 using the Jaspar database data on the NetworkAnalyst software, and the networks are presented in Figure 6D. The analysis of 45 nodes consisting in this network based on KEGG pathways identified several significant enrichments such as the MAPK signaling pathway, Wnt signaling pathway, apoptosis, cell cycle, Th17 cell differentiation, and T cell receptor signaling pathway. The results showed that FOXC1, MEF2A, and HINFP obtained the highest betweenness and degree score (represented the number of links of a single node with its binding genes) in this network. Thus, these three TFs may play a more important role in participating in POLR2G, RUVBL1, POLD1, GTF2H1, SAE1, ANAPC7, and DDB1 transcription.

### The Expression of PRPF19 was Affected by the Methylation Status and TP53 Mutation

**FIGURE 7:**
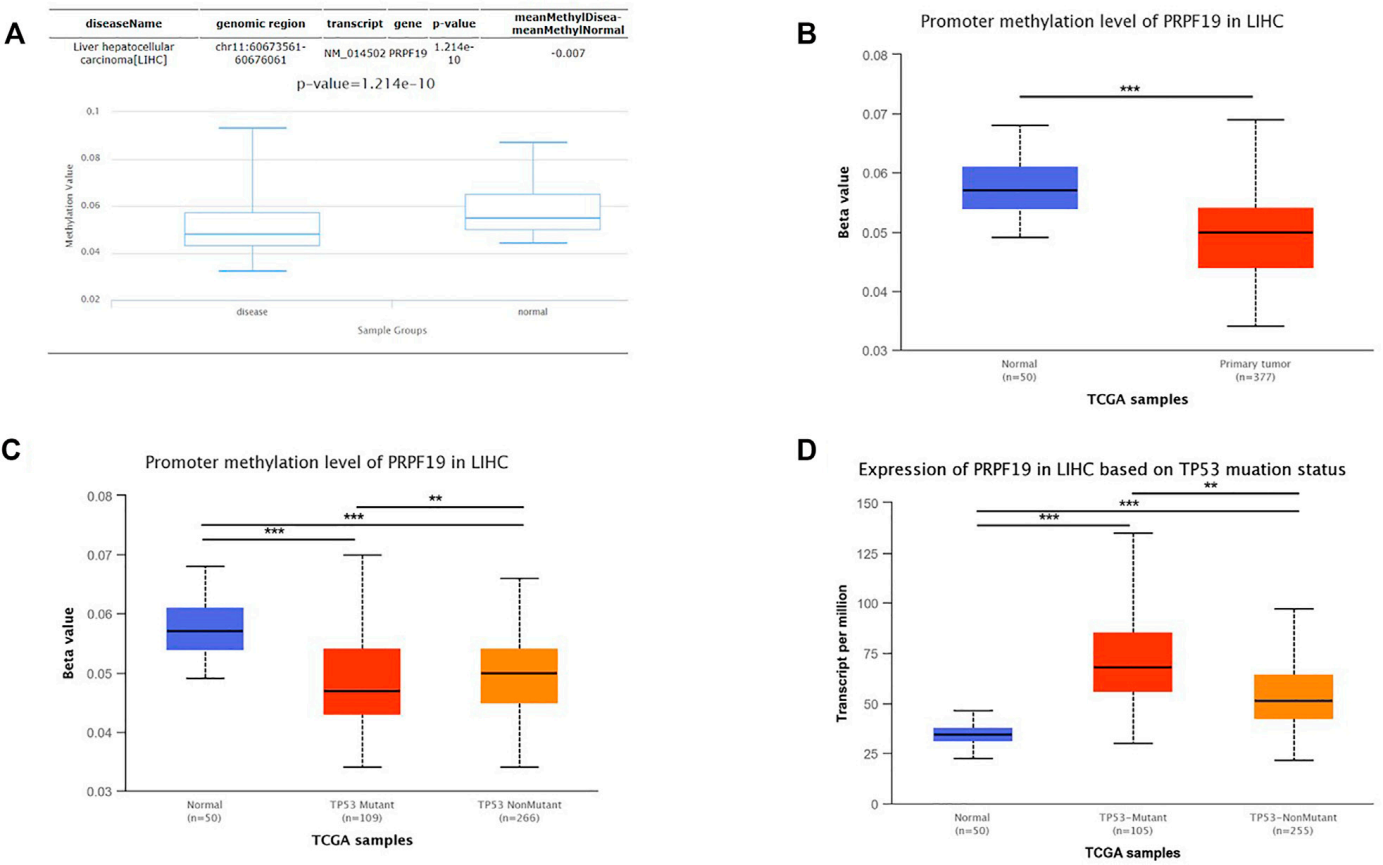
Analysis of the relationship between PRPF19 expression and promoter methylation level of PRPF19 and TP53 alteration statue. **(A)** Differential analysis of methylation value between LIHC and Control *via* DiseaseMeth. **(B)** Differential analysis of between LIHC and Control *via* UALCAN. **(C)** Correlation between promoter methylation level of PRPF19 between TP53 statue. **(D)** Differential analysis of PRPF19 expression between wild-type TP53 wild-type and mutated TP53.

To clarify underlying mechanisms of PRPF19 aberrant elevation in LIHC tissues, the relationship between the methylation status and the TP53 mutation was investigated. Considering the DNA methylation can repress gene expression (The ENCODE Project Consortium et al., 2012), we first analyzed the methylation status of PRPF19 in LIHC using the DiseaseMeth version 2.0. A significant reduction of mean methylation levels for PRPF19 was found in the tumor group compared with normal tissues (Figure 7A). We then analyzed the levels of methylation of the PRPF19 promoter using the UALCAN dataset in liver cancer. Figure 7B shows a strong inverse correlation between the PRPF19 gene expression and promoter methylation. Compared with TP53 non-mutation LIHCs, TP53 mutant LIHC demonstrated statistically significant hypomethylation in the sites of PRPF19 gene promoter (Figure 7C). As shown in Figure 7D, PRPF19 was upregulated apparently in TP53 mutant LIHC compared with TP53 non-mutant LIHC and normal tissues.

### Enrichment Analysis of Differentially Expressed Genes in the High and Low PRPF19 Groups

**FIGURE 8:**
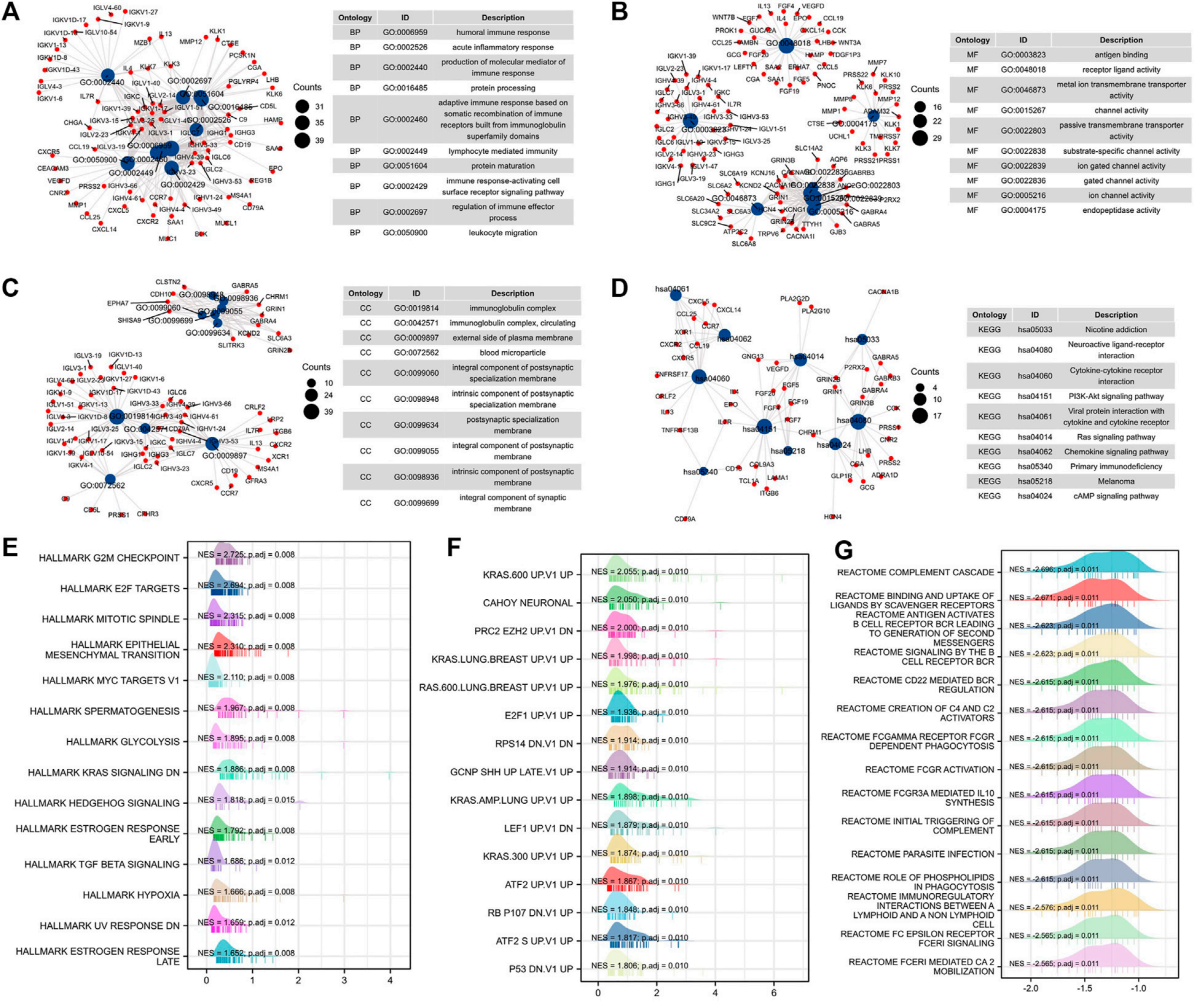
Functional enrichment analysis of PRPF19-related genes (TCGA-LIHC). **(A–D)** Gene ontology analyses, including biological process, molecular function, and cellular component were performed by clusterProfiler R package. The size of each blue spot represents the gene number represented by this term, while the red spot represents the PRPF19-related genes. Gene set enrichment analysis of PRPF19-related genes in oncology and immunology. Top 15 annotation entries of three gene sets were drawn as wave graph. **(E)** Hallmarker results of PRPF19 GSEA in LIHC. **(F)** Oncogenic signature results of PRPF19 GSEA in LIHC. **(G)** Reactome results of PRPF19 GSEA in LIHC. NES, normalized enrichment score. p.adj, adjusted *p* value.

To further study the mechanism of PRPF19-mediated progression in liver cancer, we carried out an enrichment analysis of differential genes elicited by PRPF19 from the expression profiling of LIHC-TCGA. The top ten GO terms for biological processes (BP), molecular function (MF), cell component (CC), and significantly enriched pathways are shown in Figures 8A–D.

The main enriched terms of the BP ontology are all related to cell-mediated immunity (immune response–activating cell surface receptor signaling pathway, leukocyte migration, and lymphocyte-mediated immunity). The highest-ranking CC terms agreed well with the BP result since these genes were required by the immunoglobulin complex. The MF terms included intercellular signaling interactions and exchange of materials (receptor–ligand activity, gated channel activity, channel activity, and metal ion transmembrane transporter activity) and endopeptidase activity. Moreover, pathways in cancer/immune response were enriched in KEGG, such as the PI3K-Akt signaling pathway, Ras signaling pathway, and chemokine signaling pathway. As PRPF19 was correlated with pretreatment tumor staging and tumor biology, we then performed gene set enrichment analysis (GSEA) using the MSigDB hallmark gene sets, reactome, and C6 gene sets. By GSEA, the hallmark and C6 gene sets related to cell cycle and proliferation, EMT, and KRAS (G2M checkpoint, MYC targets, E2F targets, and KRAS UP) were clearly upregulated in the PRPF19-high group (Figures 8E–G). Interestingly, tumors with high levels of PRPF19 have identified an inverse correlation with antitumor immune responses according to the reactome gene sets. Therefore, the role of the oncogene of PRPF19 in liver cancer may account for its ability to promote functions such as angiogenesis, proliferation, and immune escape.

## Discussion

Alternative splicing of eukaryotic transcripts is a fundamental mechanism that greatly expands the coding ability of a limited number of genes to generate vastly diversified proteins (Baralle and Giudice, 2017). Splicing pre-mRNA in the wrong ways is a common reason why genetic variants and pathogenic somatic mutations appear. Previous studies reported that correcting the error splicing of BRD9 (a tumor suppressor) in cancer cells bearing SF3B1 mutations effectively inhibited tumor growth (Inoue et al., 2019). Thus, some crucial components in the process of alternative splicing can be the basis for targeted cancer therapy (Robinson et al., 2019). PRPF19 is the main component of nineteen complexes (NTC) which follow the full process of translation initiation. During splicing, NTC ensures site-specific splicing for a correct balance between mRNA translation and decay (de Almeida and O’Keefe, 2015). Given the PRPF19 aberrant expression may abrogate the antitumor mechanism in humans, we did a comprehensive analysis of the clinical phenotypes and biological information relevant to this molecule in liver cancer.

PRPF19 is located on chromosome 11 and clustered within the nucleus. Until now, 117 pathogenic genes had been identified from chromosome 11 (Taylor et al., 2006). Ras family, Myc family, and other tumorigenesis genes are mainly detected at high levels in the nucleus (Yang et al., 2016; Xu et al., 2019). These findings led us to the following conjecture: for LIHC, whether the malignancy of the tumor cells is affected by changes in PRPF19 expression.

The increased PRPF19 detected in tissues and cell lines of hepatocellular carcinoma (HCC) have been reported (Yin et al., 2016); the authors also found that HCC patients with high expression of PRPF19 had a shorter OS than those with a lower expression of PRPF19. Preliminary results in our study are consistent with the aforementioned literature reports that suggest that PRPF19 expression was significantly upregulated in LIHC and associated with increased mortality. Moreover, in different subgroups, in both the advanced stage and the vascular invasion group, the survival curves of OS diverged further with the increasing length of follow-up, which suggests that high PRPF19 expression is a marker of poor prognosis for patients with liver cancer. Previous literature (Li et al., 2013) had reported that underweight (BMI <18.5 kg/m^2^) and increased AFP were associated with increased mortality from liver cancer. The overexpression of cell cycle control genes may be a critical molecular mechanism for re-activating AFP expression in liver cancer (Chen et al., 2020). As the boxplot presented in Figure 3, patients with high expression of PRPF19 also have characteristics of high-level AFP and high body weight.

In addition, pre-mRNA splicing alterations play an important role in tumor immunosuppressive microenvironment, such as driving signaling pathways altered or genes mutated (Kahles et al., 2018; Xu et al., 2021; Yu et al., 2021). In our study, a significant positive correlation exists between PRPF19 expression and several immune checkpoints as well. Furthermore, our results reveal that the main infiltrating immune cells with tumor-killing abilities were significantly reduced in the tumors with high expression of PRPF19. Tumor patients with lower abundance of MDSCs before treatment responded better to immunotherapy agents that were reported in previous literature (Chiu et al., 2017). In subsequent analysis, we found that a high expression of PRPF19 combined with high-level MDSCs was a high-risk factor for LIHC; patients harboring those traits had a poor OS. Additionally, the elevated transcript level of PRPF19 correlated with decreased response to antiPD1 treatment according to the syngeneic liver cancer mouse model data.

PRPF19 can enhance the invasive ability of neuroblastoma cells by regulated Hippo-YAP pathway, and this has been recently proposed in literature (Cai et al., 2020). We thought that the dysregulation of genes tightly related to PRPF19 may play an important role in contributing to liver cancer progression. GO and KEGG enrichment analysis showed that PRPF19-related genes were mainly enriched in immune response–activating cell terms. GSEA analysis results demonstrated that multiple cancer-related pathways (such as G2M checkpoint, MYC targets, E2F targets, and KRAS) were upregulated for LIHC with high RPPF19 expression.

Genome-wide or oncogene promoter hypomethylation are linked to tumorigenesis in multiple human malignancies (Wang et al., 2019). TP53 was a significant gene in cancer, and TP53 mutations can directly cause DNA demethylation (Samuel et al., 2016). We found that PRPF19 promoter methylation status was lower in LIHC than normal samples, and was negatively related to TP53-mutant status. Conversely, liver tumors with TP53-mutant had a higher level of PRPF19. This is likely suggested that some indirect mechanisms contribute to the aberrant PRPF19 expression in the G3-subtype of LIHC (Chidambaranathan-Reghupaty et al., 2021) (characterized by high expression of TP53 and cycle regulatory genes).

Transcription-coupled NER (TC-NER) is an RNA surveillance mechanism patrol in the cytoplasm to repair the template chain damage (Geng et al., 2020). It is well known that oncoprotein degradation can be regulated by the ubiquitin-mediated proteolysis system (UPS) (Martinez-Jiménez et al., 2020). Critically, our network analysis also supported PRPF19 as a critical gene of these signaling pathways described previously. Using networks analysis, we found that 36 TFs may be involved in regulating the function of TC-NER and UPS (participated by PRPF19).

Altogether, we provide multiple important pieces of evidence for discovering the possible causes inducing PRPF19 aberrant expressed in LIHC. These results indicated that PRPF19 would be an excellent target for LIHC prevention or treatment. Nevertheless, there are a few limitations to this research. First, our conclusions were mainly summarized based on the public datasets, and they have not yet been tested in clinical studies. Second, human liver cancer is highly heterogeneous; we performed this study without distinguishing the molecular subtypes of LIHC. Third, the regulatory mechanism of the signaling pathway involving the gene PRPF19 remains to be explored.

## Conclusion

In this study, through analysis of existing LIHC data, we proposed PRPF19 as a potential biomarker that was associated with the responses for immunotherapy, the regulation of immunosuppressive microenvironment, and can also be considered a prognostic indicator of clinical outcomes.

## Data Availability

The datasets presented in this study can be found in online repositories. The names of the repository/repositories and accession number(s) can be found in the article/Supplementary Material.
